# 

**Published:** 1887-12-31

**Authors:** 


PRICE ONE PENNY.

66, Vol. III—December 31st, 1887.

[Registered at the General Post Office as a Newspaper.]

Per Annum, 6s. 6d., post free.
Monthly Parts, 6d.; post free, 7½d.
Ditto per Ann., 7s. 6d. post free.

### THE HOSPITAL

A WEEKLY INSTITUTIONAL JOURNAL OF

